# Emerging Trends in Nanotechnology for Endometriosis: Diagnosis to Therapy

**DOI:** 10.3390/nano14110976

**Published:** 2024-06-05

**Authors:** Souvanik Talukdar, Santosh K. Singh, Manoj K. Mishra, Rajesh Singh

**Affiliations:** 1 Department of Microbiology, Biochemistry, and Immunology, Morehouse School of Medicine, Atlanta, GA 30310, USA; stalukdar@msm.edu (S.T.); sksingh@msm.edu (S.K.S.); 2 Cancer Biology Research and Training, Department of Biological Sciences, Alabama State University, Montgomery, AL 36104, USA; mmishra@alasu.edu; 3 Cancer Health Equity Institute, Morehouse School of Medicine, Atlanta, GA 30310, USA

**Keywords:** endometriosis, nanotechnology, imaging, diagnosis, treatment, magnetic hyperthermia, immunotherapy

## Abstract

Endometriosis, an incurable gynecological disease that causes abnormal growth of uterine-like tissue outside the uterine cavity, leads to pelvic pain and infertility in millions of individuals. Endometriosis can be treated with medicine and surgery, but recurrence and comorbidities impair quality of life. In recent years, nanoparticle (NP)-based therapy has drawn global attention, notably in medicine. Studies have shown that NPs could revolutionize conventional therapeutics and imaging. Researchers aim to enhance the prognosis of endometriosis patients with less invasive and more effective NP-based treatments. This study evaluates this potential paradigm shift in endometriosis management, exploring NP-based systems for improved treatments and diagnostics. Insights into nanotechnology applications, including gene therapy, photothermal therapy, immunotherapy, and magnetic hyperthermia, offering a theoretical reference for the clinical use of nanotechnology in endometriosis treatment, are discussed in this review.

## 1. Introduction

**Figure 1:**
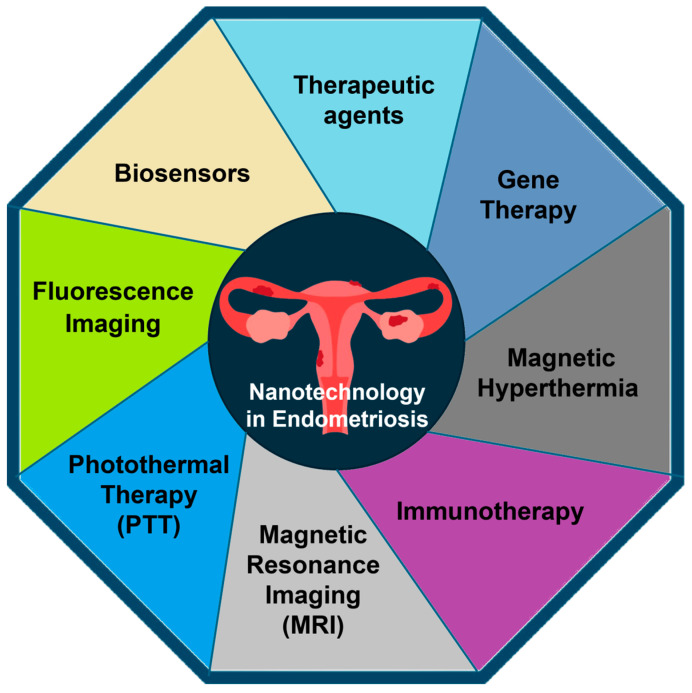
Illustration showcasing the diverse applications of nanotechnology in addressing endometriosis.

Nanotechnology, a domain of science and engineering focusing on nanoscale phenomena, is garnering significant global interest in the medical field. Numerous studies have indicated that nanoparticles (NPs) have the potential to augment traditional therapeutic methods, such as chemotherapy, and imaging techniques, like magnetic resonance imaging (MRI), for the detection (e.g., photoacoustic imaging) and treatment (e.g., photothermal therapy [PTT] and magnetic hyperthermia) of diverse diseases [1]. An overview of diverse applications of nanotechnology in addressing endometriosis is shown in Figure 1. Numerous investigations have demonstrated the potential of NPs to deliver anti-inflammatory, antioxidant, anti-angiogenic, or immunomodulating molecules to specific targets, leveraging their attributes of low toxicity, high stability, and the ability to conjugate with various biomolecules [2,3].

Endometriosis, an incurable gynecological condition, is characterized by the abnormal growth of tissue resembling the uterine lining (endometrium) outside the uterus, leading to pelvic pain and infertility in millions of individuals. This review explores the potential of nanomedicines, promising therapeutic approaches for various diseases, to revolutionize endometriosis management. We assessed existing reports and advancements in the field of endometriosis nanomedicine, providing a summary of how NP-based systems could enhance the treatment and diagnosis of endometriosis. We explored the possibility of applying fundamental principles from the established field of cancer nanomedicine to develop inventive NP-based strategies for endometriosis.

## 2. Nanotechnology in Cancer and Endometriosis

Nanoparticles, acting as modern “magic bullets”, as envisioned by Paul Ehrlich, can solubilize insoluble drugs, protect therapeutic agents from degradation, extend drug circulation time, and deliver drugs directly to disease sites, reducing systemic toxicity. This targeted approach promises more effective and safer treatments for various conditions, including cancer and infectious diseases. Numerous recent reports have suggested that nanoparticles can improve conventional therapies like chemotherapy and imaging techniques such as MRI. Currently, researchers are actively focusing on developing new treatment and imaging strategies utilizing nanomaterials, including photothermal therapy and magnetic hyperthermia. Cancer and endometriosis share similarities in terms of abnormal tissue growth, inflammation, pain, hormonal influence, and impact on fertility [4]. Both conditions involve tissue proliferation, chronic inflammation, and hormonal factors such as estrogen. They can cause pain, with endometriosis often manifesting as pelvic pain, and cancer-related pain varying depending on the affected area. Additionally, both conditions can affect fertility, with cancer treatments and endometriosis-related damage potentially impairing reproductive function. Despite these commonalities, it is essential to recognize that cancer is characterized by malignant cell growth and the potential to metastasize, while endometriosis involves the growth of benign tissue outside the uterus. Like cancer, endometriosis relies on angiogenesis for its progression, increased vasculature surrounding endometriotic lesions often found by laparoscopies [5,6]. A rising body of evidence indicates that nanomedicine has the potential to provide novel therapeutic and diagnostic strategies for endometriosis management, despite being a relatively new field in its application for imaging and treatment [4,7].

## 3. Overview of Endometriosis

Endometriosis, a condition characterized by tissue like the inner lining of the uterus growing outside the uterus, can cause frequent pain. In some rare cases these growths may extend beyond the typical pelvic organ area. Endometriosis commonly affects women and girls in their reproductive years, with prevalence ranging from 10% to 15% and occasional diagnoses even during menopause [8]. This condition, associated with pain symptoms and infertility, can significantly diminish quality of life for those affected. Typically, it impacts the ovaries, fallopian tubes, and pelvic lining.

For diagnosis, healthcare providers presently employ two primary techniques to confirm endometriosis: laparoscopy, a surgical procedure involving abdominal inflation, and advanced imaging modalities such as ultrasound and MRI; however, these noninvasive approaches are limited by cost and insufficient resolution for accurate identification of endometriotic lesions [9].

The treatment of endometriosis falls into two main categories: pharmacological and surgical. Currently, there is no specific drug to halt the disease’s progression; instead, hormonal and non-hormonal agents are used to alleviate symptoms and enhance fertility rates. Hormone therapy users often report the cessation of menstruation during treatment, posing a significant disadvantage for women seeking pregnancy.

### 3.1. Conventional Techniques

Endometriosis is conventionally treated by both surgical and pharmaceutical interventions. Surgery confirms endometriosis in 50% of women with clinical suspicions [10]. However, these measures do not constitute a lasting solution, emphasizing the urgent need for more effective diagnostics and curative interventions to alleviate the substantial physical and emotional burdens imposed by endometriosis. Nevertheless, the surgical method is acknowledged as the preferred and widely accepted standard for addressing deep endometriosis. All the guidelines encompassed by this study advocate laparoscopic surgery over laparotomy for addressing the chronic pain and infertility associated with endometriosis. This preference leads to reduced pain, shorter hospitalization periods, faster recoveries, and improved cosmetic outcomes [11,12]. On the other hand, the initial pharmacological approach recommended for addressing endometriosis involves the use of non-steroidal anti-inflammatory drugs, progestins, or combined hormonal contraceptives [13]. Nanoparticle-based therapy is still in its infancy for endometriosis. Noninvasive diagnostic methods and a definitive cure for endometriosis are urgently needed. Existing treatment options, such as pain management using anti-inflammatory medications like ibuprofen, offer only temporary relief [14], as do combined oral contraceptive pills [15].

One study involved a three-month experiment with gonadotropin-releasing hormone (GnRH) analogs to alleviate symptoms associated with endometriosis [13]. This study found that continuous GnRH application binds pituitary receptors, suppressing the pituitary-ovarian axis and reducing luteinizing hormone (LH) and follicle-stimulating hormone (FSH) levels. This leads to anovulation and lower estrogen levels due to pituitary desensitization. Furthermore, the continuous administration of GnRH contributes to endometrial atrophy, reducing the size and thickness of the endometrial lining. In summary, this comprehensive cascade of effects underscores the therapeutic impact of GnRH analogs in managing endometriosis-related symptoms.

### 3.2. Challenges

Endometriosis patients face a poor prognosis, with symptoms including bone loss, hot flashes, vaginal dryness, headaches [16], elevated testosterone levels, hirsutism, and irreversible voice deepening [13]. Furthermore, surgical procedures have potential complications, including fistulas, hemorrhages, infections, intestinal subocclusions, bladder dysfunction, or intestinal dysfunction, which can pose risks to life. The efficacy of surgery is found to be limited [17].

Nevertheless, surgical intervention offers a significant advantage over pharmaceutical alternatives due to its capacity to not only improve fertility but also deliver concurrent pain relief. Due to its ability to boost fertility and relieve discomfort, surgery is better than pharmaceuticals. Surgery targets the underlying causes of infertility, such as endometriosis, unlike pharmaceuticals. Fertility is improved by surgically correcting anatomical anomalies, removing impediments, or increasing reproductive organ performance. Surgery can also relieve pain by directly targeting the key factors, providing a comprehensive approach that goes beyond symptom management for fertility improvement and pain relief.

While surgical treatment for endometriosis carries notable advantages, it is vital to acknowledge the potential for complications during or after procedures and the possibility of lesion recurrence. Statistics indicate that approximately 27% of patients may require multiple surgical interventions. Despite the benefits offered by surgery, the occurrence of complications and the need for repeated procedures underscore the existing limitations of this approach. Hence, there is an urgent need for improved treatment approaches to manage endometriosis better, decrease complications, and prevent lesion recurrence. This emphasizes the necessity for continuous research and innovation to enhance treatment strategies for those affected by endometriosis [2].

## 4. Applications of Nanotechnology in Endometriosis

In recent years, the nanomaterial treatment approach has significantly impacted endometriosis. These strategies involve diagnosing and treating endometriosis using nanotechnology approaches called nanotherapies, which include controlled drug delivery, magnetic hyperthermia, and NP-mediated photothermal therapy, etc. [18]. Endometriosis has shown similar pathophysiological characteristics to cancer, such as angiogenesis reactive oxygen species (ROS) production. Consequently, certain fundamental principles of applied nanotechnology in cancer research can be extended to, or have already been employed in, the context of endometriosis [2]. Some of the applications are summarized below.

### 4.1. Diagnosis

Certain NPs, particularly those constructed from materials such as iron oxide or gold, possess inherent characteristics that enable them to enhance the contrast agents used in magnetic or optical imaging methods. Alternatively, some NP systems, such as fluorescent dyes, encapsulate molecules to achieve heightened contrast between afflicted and healthy tissues or cells. NP-enhanced imaging involves a straightforward injection or infusion of the NP material and noninvasive imaging of the tissues of interest [19]. This stands in stark contrast to invasive laparoscopy techniques.

Despite these advantages, NP-enhanced imaging relies on contrast agent delivery to targeted lesions. Delivery precision is crucial for ensuring accurate imaging and diagnostic outcomes. NP contrast agents may be retained in the body with uncertain long-term effects. Clinicians and patients must assess the benefits of improved imaging against the potential risks associated with the prolonged presence of these NPs in the body. Thus, when considering NP contrast agents for lesion detection in medical practice, it is crucial to weigh the pros and downsides.

#### 4.1.1. Fluorescence Imaging

The process of diagnosing endometriosis can be time-consuming and is hindered by the complex nature of the mechanisms underlying its various etiologies. Currently, the most dependable diagnostic method involves laparoscopy and histological confirmation. However, it is worth noting that this procedure, while reliable, comes at a significant cost and poses potential surgical risks [20]. The successful identification and removal of endometriotic lesions during laparoscopy relies on the surgeon’s skill in discerning between healthy tissue and endometriotic lesions. This task is particularly challenging because endometriotic lesions can manifest in various forms and exhibit diverse colorations, emphasizing the need for precision and expertise during the surgical procedure [21]. Certain lesions exhibit pigmentation, which makes them readily identifiable during surgery. Conversely, non-pigmented lesions typically appear pale and pose a greater challenge for identification. Hence, employing real-time surgical fluorescence imaging techniques can potentially improve the identification of non-pigmented lesions.

For effective lesion visualization using fluorescence, targeted accumulation of fluorescent agents within specific cellular structures is crucial. One notable molecule, protoporphyrin IX (PPIX), tends to accumulate within cancer cells after administering aminolevulinic acid (ALA). This process, involving aminolevulinic acid, is pivotal in facilitating fluorescence visualization of lesions, especially in cancerous tissues like malignant gliomas [22].

Due to the shared characteristics between cancer and endometriotic lesions, the introduction of exogenous ALA was hypothesized to trigger the intracellular buildup of fluorescent PPIX within these lesions. Studies have consistently shown that treatment with 5-ALA results in selective accumulation of PPIX in endometriotic lesions, as opposed to the neighboring normal peritoneum [22,23]. These findings reveal a heightened level of porphyrin fluorescence in active peritoneal endometriosis compared with the adjacent normal peritoneum. This suggests that exogenous ALA treatment could target endometriotic lesions by accumulating fluorescence PPIX, offering diagnostic and therapeutic potential [23,24]. While exogenous ALA administration enhances fluorescence in endometriosis, its efficacy is confined solely to nonpigmented lesions. Consequently, this method has detected no fluorescence in pigmented or ovarian endometriosis [24].

Due to limitations, researchers have sought a more accurate technique for fluorescence-guided surgery, exploring options such as using near-infrared (NIR) dyes and light to diagnose endometriosis [25]. NIR light possesses advantageous characteristics, including superior tissue penetration and reduced scattering without causing tissue damage, even with prolonged exposure. In contrast, visible light struggles to penetrate deeply into blood and tissue due to energy depletion from absorbance and scattering, limiting visibility to surface features only. Furthermore, the NIR window, spanning from 650 to 900 nm, aligns with local absorption minima for major biomolecules like deoxyhemoglobin, oxyhemoglobin, water, and lipids [26]. Given these properties, NIR fluorescence-guided surgery, leveraging NIR dyes and activation light, emerges as a promising strategy for real-time demarcation of endometriotic lesions. Recurrence and harm to healthy tissues are both reduced with this approach. The unique advantages of NIR light, including enhanced tissue penetration and selective absorption properties, make it a promising diagnostic tool for endometriosis [26].

For NIR imaging, the FDA-approved tricarbocyanine dye indocyanine green (ICG) is commonly used as a contrast agent [27]. Due to its affinity for plasma proteins, at least 95% of the dye remains intravascular despite its widespread medical use. This characteristic may impede its effective diffusion into specific target sites. Additionally, ICG in solution tends to aggregate and form oligomers, even at low concentrations. This aggregation propensity can pose challenges in specific applications [28,29].

In another study, researchers explored using NPs to deliver NIR fluorescent dyes to endometriotic lesions systemically [30]. This study employed a sophisticated approach by introducing “activatable” silicon naphthalocyanine-loaded polyethylene glycol-polycaprolactone NPs (SiNc-NP), demonstrating efficient accumulation in endometriotic lesions upon systemic administration. SiNc was selected based on its superior fluorescence efficiency and photostability compared to alternative contrast agents like ICG. While SiNc shows promise as a contrast agent for fluorescence-guided surgery, its application is impeded by limited water solubility, measuring less than 1 nanogram per milliliter (<1 ng mL^−1^) [31]. This innovative approach involving NP-based delivery systems opens paths for enhancing the targeted delivery of NIR fluorescent dyes to endometriotic lesions, potentially overcoming solubility challenges and improving contrast agents’ efficacy in real-time imaging during surgical procedures [30,32,33,34].

#### 4.1.2. Magnetic Resonance Imaging (MRI)

Utilizing imaging modalities such as MRI has proved to be instrumental in diagnosing endometriosis by enabling the visualization of lesions. This imaging technique not only aids in diagnosis but also serves as a valuable tool for researchers and clinicians, offering insights into the origin and development of the disease. To augment the precision and sensitivity of these imaging methods, NP contrast agents are coated with ligands designed to bind specifically to endometrial cells or related reproductive tissue. This targeted approach enhances imaging capabilities, ensuring more sensitive and accurate visualization and localization of endometriosis, thereby providing a deeper understanding of the disease [35].

Numerous studies have suggested that the signal ratios between lesions and the surrounding background in MRI can be elevated by utilizing T2-negative contrast agents such as magnetic iron oxide NPs. Lee et al. introduced a novel diagnostic instrument for assessing experimentally induced endometriosis [36]. The study focused on the contribution of ultrasmall superparamagnetic iron oxide (USPIO) particles. Falling within the category of superparamagnetic iron oxide NPs with a size of less than 50 nm, USPIOs are part of the SPIO family. Notably, this class of NPs has obtained approval from both the US FDA and the European Commission for use as a contrast agent in MRI. This recognition underscores their safety and suitability for enhancing contrast in MRI scans, highlighting their potential significance in medical imaging applications [37].

In a recent investigation, researchers successfully engineered magnetic iron oxide (Fe_3_O_4_) NPs for utilization as negative contrast agents in T2-weighted MRIs of endometriosis. These NPs were deliberately designed with high relaxivity, low toxicity, and outstanding contrast enhancement. Notably, the study aimed to address the specificity of targeting endometriotic cells. To achieve this, the Fe_3_O_4_ NPs were subjected to modification with hyaluronic acid (HA). This strategic modification improved NP targeting of endometriotic cells with overexpressed CD44 receptors. This approach leverages the favorable imaging properties of Fe_3_O_4_ NPs and tailors their surfaces to facilitate precise targeting, making them a promising tool for enhanced and targeted MRI imaging of endometriosis [38]. Further, endometriosis was induced in rats through surgical means by autologous transplantation. Four weeks after the surgical procedure, rats received intravenous (IV) HA-Fe_3_O_4_ NPs and MRI imaging. The lesion walls darkened in animals injected with HA-Fe_3_O_4_ NPs after 2 h, especially during turbo spin-echo fat-suppressed T2-weighted MRI. The presence of NPs within the lesions was verified through quantitative assessment of iron concentrations and confirmed by staining excised tissues with Prussian blue. Remarkably, ectopic endometrium had more NPs than eutopic endometrium. This discrepancy underscored the efficacy of utilizing CD44 as a marker for targeted lesion identification, further validating the precision of the NP delivery system.

#### 4.1.3. Biosensors

Understanding the fundamentals and benefits of sophisticated tools over traditional sensors is important. Biosensor assays, for instance, utilize a reporter, often an antibody labeled with a fluorophore, to identify the presence of a specific antigen in serum. NP-based systems play a pivotal role in enhancing sensitivity and signal-to-noise ratios compared with conventional approaches. They achieve this by amplifying the readout, such as by elevating the fluorescence quantum yield or strengthening binding to the targeted molecule [39].

In addition to fluorescence-based assays, this amplification affects other detection modalities, such as absorbance-based techniques, electrical measurements, and many more. Incorporating NPs into these sensing technologies provides a multifaceted enhancement, increasing precision and reliability in detecting and quantifying specific molecules. These detection method improvements illustrate the need to use and comprehend modern technologies for more efficient analytical processes.

Furthermore, identifying biomarkers is a promising noninvasive avenue for diagnosing endometriosis. Research shows that endometriosis patients have a microbial composition in their peritoneal fluid and feces which is distinct from those without the condition [35]. This suggests that analyzing these microbial profiles could help to diagnose and increase the understanding of endometriosis [35].

In a separate study, individuals with endometriosis were found to exhibit unusually high cancer antigen 125 (CA125) concentrations in their serum, surpassing the normal threshold of 35 units per mL. Thus, CA125 assays serve as another diagnostic method for identifying endometriosis [39]. CA19-9 has surfaced as a more favorable biomarker than CA125 due to its greater sensitivity and specificity [20,40]. Therefore, researchers have created a bio-nanocomposite to detect CA19-9 in serum. This sensor is composed of multi-walled carbon nanotubes (MWCNT) and magnetite (Fe_3_O_4_) NPs dispersed in chitosan (CS). To enhance its functionality, the nanocomposite was coupled with anti-CA19-9 antibodies. This innovative approach holds promise for sensitive and specific detection of CA19-9, showcasing the potential of bio-nanocomposites for advancing biomarker sensing technologies [39]. Harada et al. found that serum cancer antigen 19-9 (CA19-9) levels are better predictors of endometriosis severity than cancer antigen 125 (CA125) levels. Elevated CA19-9 levels in the serum of endometriosis patients, associated initially with colorectal carcinoma cells, suggest its role as a biomarker. The research indicates that heightened CA19-9 levels may signify potential metastasis in endometriosis.

Additionally, the study explores the presence of CA19-9 in ovarian chocolate cysts’ glandular epithelial cells, suggesting a possible contribution to endometriosis progression. Serum CA19-9 levels were elevated in advanced endometriosis stages (III and IV), though its sensitivity was lower than CA125, especially in earlier stages [39,41]. The study proposes reevaluating the cutoff value for CA19-9 levels, advocating for a new range (20 to 25 IU/mL) to enhance sensitivity without compromising specificity. The study concludes that serum CA19-9 and CA125 can indicate endometriosis severity; however, further studies are needed to identify an optimal cutoff value [39,42].

Moreover, gold NPs (AuNPs) are appealing for use as immunosensors due to their rapid synthesis, excellent conductivity, and facile antibody labeling for biomolecule detection. With its mechanical strength, large surface area, and high electrical conductivity, graphene is another material well-suited for immunosensing applications. Graphene oxide’s (GO’s) interaction with metal NPs like AuNPs creates efficient electrochemical sensors [43]. Sangili et al. developed a label-free immunosensor by modifying glassy carbon electrodes (GCE) with AuNP/reduced-GO, enhancing it with anti-CA125 antibodies through a layer-by-layer assembly process [44]. A suggested immunosensor for assessing glycoprotein haptoglobin (HP) on an Au/rGO hybrid film was proposed.

### 4.2. Treatment

#### 4.2.1. Nanomaterials and Therapeutic Agents

For endometriosis treatment, therapeutic strategies have been developed based on single agents capable of targeting one or multiple therapeutic pathways. Nanomaterials, commonly employed as drug delivery carriers, can also serve as therapeutic agents due to their unique characteristics. For instance, Chaudhury et al. utilized cerium oxide NPs (CNPs or nanoceria) as a therapeutic intervention to mitigate ROS levels and angiogenesis within the peritoneal cavity of mice induced with endometriosis [45].

Oxidative stress (OS) is implicated in various diseases such as cancer, endometriosis, and cardiovascular disease. Cerium oxide NPs (nanoceria) have emerged as crucial in treating oxidative stress-related conditions. Thus, Chaudhury et al. [45] explored the potential of nanoceria in endometriosis treatment. Compared to control mice, nanoceria markedly diminished endometrial lesions by reducing oxidative stress markers and angiogenesis in mice with induced endometriosis. This suggests that nanoceria holds promise as a therapeutic option for endometriosis.

Oxidative stress contributes to the pathophysiology of endometriosis by inducing a systemic inflammatory response within the pelvic cavity. This inflammation may elevate local aromatase activity and estrogen levels. Like tumors, reactive oxygen species (ROS) have been demonstrated to enhance the proliferation rate of endometriotic cells [46,47,48]. The theory proposes that menstrual reflux contributes to an imbalance in ROS and antioxidant defenses due to the deposition of erythrocytes, apoptotic endometrial tissue, and cellular debris in the peritoneal cavity. Therefore, therapeutic strategies, including those centered on nanomaterials, increasingly focus on addressing oxidative stress phenomena.

The major limitations of endometriosis treatment are poor stability, low biological activity, and targeting. Nanomaterials, serving as drug delivery carriers, can address these drawbacks. Epigallocatechin gallate (EGCG) and doxycycline (DOX) are known for their antioxidant and antiangiogenic properties, and they are applied in endometriosis treatment [49]. However, their instability hampers therapeutic use. Poly (lactic-co-glycolic acid) (PLGA) is an ideal candidate for drug delivery, enhancing stability and bioavailability. Singh et al. synthesized single drug-loaded PLGA NPs (EGCG NPs, DOX NPs) and dual drug-loaded NPs (DOX-EGCG NPs) for treating endometriosis in mice. The dual drug-loaded NPs proved more effective in reducing oxidative stress, angiogenesis, and MMP activity than single drug-loaded NPs [50]. Copaiba oleoresin (CPO), a natural product from the *Copaifera* plant, is used to treat endometriosis by inhibiting the proliferation of human endometrial stromal cells [51]. PLGA NPs and CPO were investigated for their impact on human endometrial stromal cells, demonstrating a reduction in cell viability in the ectopic and eutopic endometriotic lesions of patients with endometriosis [52]. Boroumand et al. synthesized nanofibers loaded with curcumin using poly ε-caprolactone (PCL) and polyethylene glycol (PEG). The investigation aimed to assess the enhanced release of curcumin both in vitro and in vivo [53]. The findings revealed that curcumin-loaded PCL-PEG nanofibers exhibited an approximately 50% release of curcumin over 30 days in vitro. Moreover, the nanofibers loaded with curcumin demonstrated significant improvements in endometriosis, manifested by histological changes such as smaller endometrial glands, stroma, and inflammatory cell infiltration.

#### 4.2.2. NP-Mediated Photothermal Therapy

Photothermal therapy (PTT) employs light-absorbing agents like NPs to convert optical energy into heat, selectively destroying targeted cells through protein denaturation and membrane damage. Commonly using NPs such as gold nanorods or carbon nanotubes, PTT relies on photothermal conversion, where absorbed laser light generates heat, inducing localized hyperthermia [54]. PTT is suitable for cancer treatment, offering a minimally invasive approach with precise heat control [55]. NPs are designed to absorb light in the NIR region, optimizing tissue penetration. The therapy’s effectiveness depends on factors like the choice and concentration of photothermal agents and laser parameters. Ongoing research aims to optimize PTT, expanding its applications and enhancing its clinical efficacy [56,57,58]. Recent advances in nanomedicine have accelerated the swift evolution of light-induced theragnostics. This progress is grounded in the use of versatile nano-agents endowed with diverse light-induced functionalities, encompassing the conversion of NIR to visible light, as well as photodynamic therapy (PDT) and PTT. Phototherapy utilizing NP-based systems involves systemic or local nanosystems and NIR laser irradiation within the wavelength range of 700 nm to 1350 nm [54,56]. An ideal nanosystem for this purpose should exhibit superior characteristics, including heightened absorption efficiency, efficient conversion of light into heat (photothermal conversion efficiency), minimal toxicity, and a commendable capacity for precise targeting.

This technique requires an effective equilibrium between these attributes for effective and safe therapy. Gold NP (AuNP)-mediated PTT, extensively explored as a standalone cancer treatment, has recently demonstrated enhanced therapeutic outcomes when combined with other imaging and treatment modalities due to the tunable optical properties of AuNPs [59,60]. Of all the nanomaterials employed in PTT, AuNPs have gained the broadest utilization. AuNPs have emerged as the predominant nanomaterials in PTT due to their unique ability to undergo a collective, coherent oscillation process known as localized surface plasmon resonance, facilitated by their unique interactions with light. Guo et al. achieved similarly promising outcomes by creating a targeted PTT system for endometriosis using hollow, gold nanoshells (HAuNS) coated with TNYL peptides. These peptides bind to EphB4 receptors, which are overexpressed in endometriosis lesions [61].

**Figure 2:**
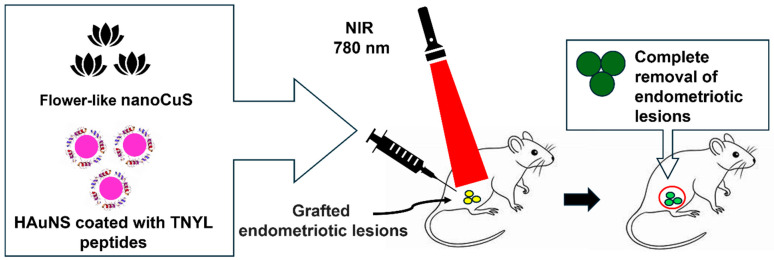
Overview of photothermal therapy (PTT) using flower-like nano copper sulfide (CuS) and hollow gold nanoshells (HAuNS) coated with TNYL peptides for treating endometriosis by near-infrared (NIR) laser irradiation.

A study found that flower-like nano copper sulfide NPs had a 50% higher photothermal conversion efficiency than hexagonal sulfide NPs, highlighting the significance of NPs’ morphology in influencing their performance [62]. Moses et al. devised a nano platform utilizing silicon naphthalocyanine (SiNc) dye and polymeric NPs for real-time NIR fluorescence imaging and PTT [30]. SiNc NPs are particularly well-suited to use in photothermal therapy due to their robust absorption of NIR light, generating high NIR fluorescence and heat under relatively low-power NIR light exposure. Addressing the poor solubility and tendency to aggregate in aqueous environments, the researchers employed a solvent evaporation method to encapsulate SiNc molecules within the hydrophobic core of [methoxy poly (ethylene glycol)-*b*-poly(ε-caprolactone)] (PEG-PCL) polymer NPs. The primary objective was to achieve enhanced contrast in fluorescence imaging of endometriotic lesions. However, it was discovered that these materials can generate heat upon exposure to NIR light, opening possibilities for their application in the combined imaging and PTT of endometriosis. The efficacy of PTT with this system was assessed through both in vivo and in vitro examination [30]. Figure 2 depicts an overview of the use of PTT in endometriosis using NIR laser irradiation.

The results show that NP-based PTT can treat endometriosis, indicating the potential of repurposing NPs. Nevertheless, limitations exist due to insufficient tissue penetration and the high levels of light intensity required to activate currently available photosensitizers. Therefore, Moses et al. [30] concluded that PTT is a viable, effective, and safe endometriosis treatment warranting additional study.

#### 4.2.3. Immunotherapy

Immunotherapy is a medical treatment that harnesses the body’s immune system to combat diseases, particularly for cancer and certain autoimmune disorders. NP immunotherapy for endometriosis modulates and enhances the immune response against endometrial lesions. This innovative approach aims to harness the unique properties of NPs to stimulate or regulate the immune system, providing a targeted and effective strategy for managing endometriosis. So far, research has focused on macrophages or T cells. Macrophages are significant due to their ability to identify and eliminate endometrial cells from the peritoneal cavity. The polarization of these macrophages plays a crucial role, with a heightened presence of M2 macrophages fostering fibrosis and angiogenesis, thereby contributing to the development of endometriosis. Studies have indicated that transplanting M1 macrophages can decrease the growth of endometrial lesions, while transplantation with M2 macrophages encourages lesion development. Numerous studies have linked endometriosis to immunologic variables, including increased CD4^+^CD25^+^ regulatory T cells. To inhibit the activation of these regulatory T cells, anti-CTLA-4 (cytotoxic T lymphocyte antigen-4) has been employed as an immune checkpoint inhibitor [63,64]. Leveraging the versatility of PLGA-based drug delivery systems across various ailments, researchers synthesized NPs comprising PLGA/anti-CTLA-4 to explore their potential in endometriosis treatment [65]. The findings indicated that PLGA/anti-CTLA-4 significantly reduced the percentage of CD4^+^CD25^+^ Treg cells in peritoneal fluid in a mouse model of endometriosis. Additionally, PLGA/anti-CTLA-4 curtailed the proliferation and invasion of ectopic endometrial cells by suppressing the secretion of IL-10 and TGF-β by CD4^+^CD25^+^ Treg cells.

**Figure 3:**
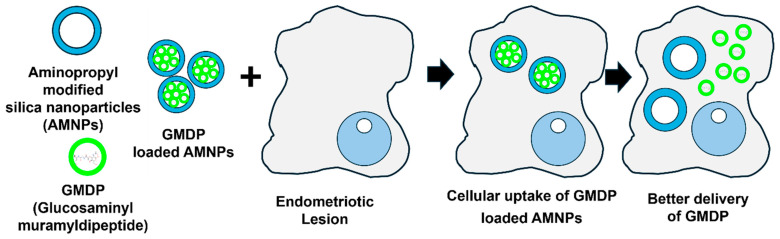
Aminopropyl-modified silica NPs (AMNPs) exhibited heightened activation of diverse pattern recognition receptors when utilized to deliver the immunomodulatory drug GMDP (glucosaminyl muramyl dipeptide).

Another study manipulated macrophages. Two distinct nanocarriers, unmodified mesoporous silica NPs (UNPs) and aminopropyl-modified silica NPs (AMNPs), were investigated for delivering the immunomodulatory drug GMDP (glucosaminyl muramyl dipeptide) to endometriosis-derived macrophages [66]. Both types of particles exhibited substantial cellular uptake with low cytotoxicity. However, when employed to deliver GMDP, the AMNPs displayed more potent activation of various pattern recognition receptors (including CD36, CD204, NOD2, and RAGE) in treated peritoneal macrophages than free GMDP or GMDP-UNPs. Furthermore, GMDP-AMNPs increased the expression of MMP-9 (matrix metalloproteinase-9), aiding macrophages in breaking down the extracellular matrix of targeted cells for phagocytosis [66]. An overview of immunotherapy using AMNPs to deliver GMDP in endometriotic lesions is shown in Figure 3.

Li et al. [67] opted for intraperitoneal injection of M1-like macrophages (M1NVs). Their method targeted the peritoneal cavity rather than the extracellular vesicles (EVs), which tend to accumulate in the liver, lungs, and spleen due to their high macrophage content in these organs, potentially causing organ damage. The peritoneal cavity serves as the primary site for endometriosis lesions and is rich in macrophages. When M1NVs were injected into the peritoneal cavity, they were promptly engulfed by macrophages, with minimal absorption into the bloodstream. These results demonstrated that intraperitoneal injection of M1NVs did not lead to accumulation in the organs. Consequently, the authors proposed that intraperitoneal injection may be suitable for treating endometriosis with M1NVs [67].

#### 4.2.4. Magnetic Hyperthermia

Although PTT holds potential for managing endometriosis, it encounters a challenge due to the restricted tissue penetration of NIR light, typically limited to less than a few centimeters. This limitation hinders the effectiveness of PTT in treating deeper-seated lesions. However, an alternative approach that overcomes these depth constraints is magnetic hyperthermia. This technique uses an alternating magnetic field (AMF) to stimulate magnetic NPs, inducing heat production and showing great promise as a targeted cancer therapy [68,69].

Unlike PTT, magnetic hyperthermia does not rely on light penetration and can effectively target deep-seated endometriosis lesions. The application of an AMF provides a versatile and noninvasive method for generating localized heat, offering a promising alternative therapeutic intervention in endometriosis cases where the limitations of PTT may pose challenges [2].

**Figure 4:**
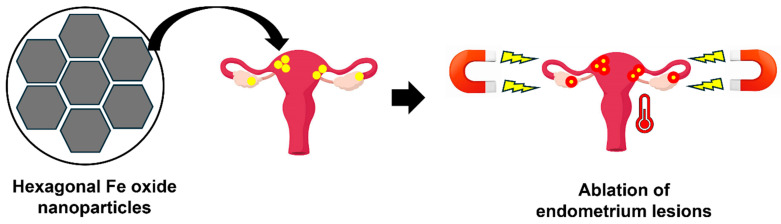
Hexagonal iron oxide NPs show enhanced heating efficiency with low cobalt content. Representative figures show precision magnetic hyperthermia for endometriosis, which peptides that bind to VEGFR-2 (KDR) enable.

Park et al. designed hexagonal iron oxide NPs infused with a small cobalt content to enhance heating efficiency [2]. These NPs were then enclosed in nanocarriers based on poly(ethylene glycol)-block-poly(ε-caprolactone) (PEG-PCL) and modified with peptides to specifically target VEGFR-2 (also known as KDR), facilitating precise magnetic hyperthermia for endometriosis treatment [2]. The choice of targeting VEGFR-2/KDR stemmed from its elevated expression in endometriosis lesions compared to the eutopic endometrium. The efficacy of the KDR-targeted magnetic NPs (MNs) was assessed through both in vitro and in vivo experiments. The cells exposed to KDR-MNs exhibited faster heating in the presence of an AMF due to enhanced cellular binding. This study proposes that targeted magnetic hyperthermia is a potential non-surgical and safe approach for eliminating deeply rooted endometriosis lesions. Figure 4 shows the schematic of magnetic hyperthermia using hexagonal iron oxide NPs for endometriosis treatment.

#### 4.2.5. Gene Therapy

Gene therapy aims to alter genetic material to treat or prevent diseases. This can involve the addition of a functional gene, the correction of a faulty gene, or the removal of a harmful gene [70]. Depending on the desired outcome, gene therapy can allow the generation of functional proteins or the suppression of detrimental gene expression in target cells post-transfection. These effects can be accomplished through diverse methods, such as the delivery of plasmid DNA containing genes of interest. These genes can be expressed transiently or stably into the genome [71].

Zhao et al. described a straightforward and efficient approach for establishing a stable vector system with varied structure–activity relationships [72]. Accordingly, the micelles exhibited notable internalization into rat tissue and efficient accumulation within ectopic endometriosis lesions. Chitosan micelles/pigment epithelium-derived factor (CSO-SA/PEDF) effectively inhibits ectopic endometrial proliferation, leading to atrophy and degeneration while preserving normal reproductive organ function. These characteristics render CSO-SA vectors highly promising for extended exploration. The investigation focused on a thorough characterization of polymer properties, emphasizing their potential for targeted gene therapy in endometriosis and a deeper understanding of the underlying treatment mechanisms.

**Figure 5:**
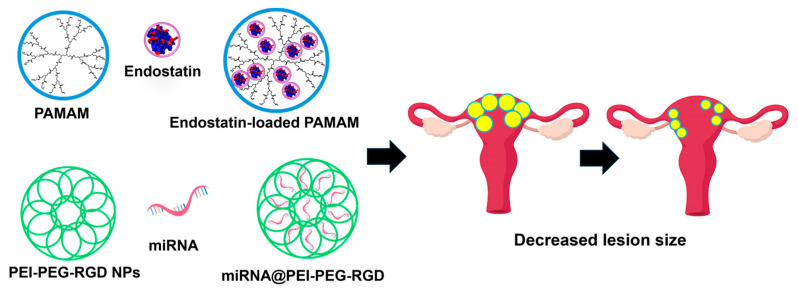
Schematic demonstration of gene therapy for treating endometriosis involving the utilization of endostatin-loaded PAMPAM dendrimers alongside polyethyleneimine-polyethylene glycol-arginine-glycine-aspartic acid (PEI-PEG-RGD) carrying miR-200c mimic RNA (miRNA@PEI-PEG-RGD). These approaches resulted in a notable reduction in lesion size.

Another study used polyamidoamine (PAMAM) dendrimers to deliver endostatin-containing plasmids, a potent angiogenesis inhibitor [73]. Upon direct injection into GFP-expressing subcutaneous endometriosis lesions in nude mice, the PAMAM dendrimers loaded with endostatin plasmids significantly decreased lesion size in experimental mice compared to control mice (depicted in Figure 5). This was assessed by analyzing GFP fluorescence intensity on days 15, 20, 25, and 30 and measuring lesion weight on day 30. Additionally, the endostatin-loaded PAMAM demonstrated a reduction in microvessel density, as observed in immunohistochemistry analysis.

Liang et al. [74] designed NPs consisting of polyethyleneimine-polyethylene glycol-arginine-glycine-aspartic acid (PEI-PEG-RGD), carrying miR-200c mimic RNA (miRNA@PEI-PEG-RGD). They investigated the impact of these NPs on ex vivo human endometriotic stromal cells (HESC’s) and in a subcutaneous rat model of endometriosis. After transfection, both proliferation and migration of HESC’s were markedly inhibited compared to control groups. The introduction of an miR-200c inhibitor significantly increased HESC proliferation and migration. MiR-200c demonstrated a dose-dependent downregulation of MALAT1, and treatment with miRNA@PEI-PEG-RGD led to a significant reduction in several mesenchymal markers in HESCs. In a rat model, direct intra-lesional injection of miRNA@PEI-PEG-RGD NPs led to a substantial decrease in endometriotic cyst volume after 20 days in experimental groups as opposed to control groups (saline, empty delivery vehicle, and scrambled miRNA) [74].

## 5. Conclusions

While the use of nanomedicine for endometriosis is in its early stages, emerging reports suggest that NP-based strategies can revolutionize diagnosing and treating the condition. Notably, existing evidence indicates that NP platforms validated in cancer studies exhibit similar biodistribution profiles and efficacy in rodent models of endometriosis, offering potential applications in detection and treatment. However, endometriosis’s unique etiology and pathogenesis warrant extensive investigation into the mechanisms of NP accumulation in ectopic endometrium. Understanding these mechanisms is crucial for designing efficient NPs tailored to the specific features of the disease.

Nanotechnology has gained traction in endometriosis treatment due to its biocompatibility, high targeting abilities, and low toxicity. Nanomaterials serve as effective delivery carriers for drugs, enhancing treatment efficacy. Various applications, including traditional therapies, gene therapy, immunotherapy, photothermal therapy, and magnetic hyperthermia, highlight nanotechnology’s pivotal role in addressing the challenges posed by endometriosis. The absence of a cure and limited lasting treatments necessitates innovative approaches, especially considering the delays in diagnosis and the challenges in characterizing disease severity. Researchers are increasingly exploring nanotechnology-based solutions to improve treatment and diagnostics for endometriosis, leveraging the advantages of biocompatible and easily modifiable NPs. Table 1 summarizes the technologies discussed in this review, emphasizing the progress in developing noninvasive diagnostic tools and effective treatments for endometriosis. The unique characteristics of nanomedicines, including hyperthermia, drug delivery, gene therapy, and immunotherapy, show substantial promise in transforming patient care. Additionally, advancements in sensors and imaging agents support earlier detection, addressing critical unmet needs in endometriosis management.

## 6. Future Scope

In the context of endometriosis treatment, assessing the biological safety of nanomaterials is crucial. While some studies suggest no adverse effects on normal endometrial cells and animal weights, the impact on other tissues, long-term health, and offspring remains uncertain. Further research on nanomaterial safety is needed to optimize their performance for endometriosis treatment. Despite significant effects in animal models, clinical applications of nanotechnology for endometriosis lack extensive research and financial support. Challenges include the difficulty in predicting patient prognosis, limited clinical trials, and a need for more validated biomarkers like miRNA and siRNA for noninvasive diagnostics. Imaging techniques, such as fluorescence and photoacoustic imaging, hold promise, especially with targeted NPs, for improved diagnostic accuracy. Future treatment advancements require innovative nanotherapy designs, accurate animal models (preferably primate models), safety testing, and evaluation in clinically relevant settings, to enhance translatability and efficacy in endometriosis patients. The effectiveness of NP-based platforms demonstrated in rodent models emphasizes the urgency of assessing their efficacy in more clinically relevant animal models, like monkeys, and eventually in human clinical trials. Understanding the heterogeneity of passive targeting in endometriosis patients is crucial in determining whether results from rodent models align with clinical outcomes. If not, focusing on primate models could efficiently translate findings to clinical practice and streamline the development of effective nanoplatforms for endometriosis treatment.

## Figures and Tables

**Table 1:** Summary of nanomedicines for imaging and treating endometriosis.

Application	Nanomaterial	Cargo	References
MRI	Iron oxide NPs	N/A	Lee et al., 2012 [36]
			Zhang et al., 2014 [38]
Fluorescence imaging	PEG-PCL polymeric NPs	NIR dye (SiNc)	Moses et al., 2020 [30]
Photothermal therapy	Hollow gold nanospheres	N/A	Guo et al., 2017 [61]
	PEG-PCL polymeric NPs	NIR dye (SiNc)	Moses et al., 2020 [30]
Therapeutic agent	Nanoceria		Chaudhury et al., 2013 [45]
Drug Carrying NPs	PLGA	Epigallocatechin gallate and doxycycline	Singh et al., 2015 [50]
	PLGA	Copaiba oleoresin	de Almeida Borges et al., 2018 [52]
	PEG and PCL nanofibers	Curcumin	Boroumand et al., 2019 [53]
Immunotherapy	PLGA	Anti-CTLA-4	Liu et al., 2017 [65]
	M1NVs (nanovesicles derived from M1 macrophages)		Li et al., 2021 [67]
	Aminopropyl modified silica NPs (AMNPs)	GMDP (glucosaminyl muramyl dipeptide)	Y. Antsiferova et al., 2013 [66]
Magnetic hyperthermia	Hexagonal iron oxide NPs coated by PEG-PCL	KDR	Park et al., 2022 [2]
Gene Therapy	CSO-SA	PEDF	Zhao et al., 2012 [72]
	(CSO-PEI) HA	AQP2-siRNA	Zhao et al., 2016 [75]
	PAMAM	Endostatin	Wang et al., 2014 [73]
	PEI–PEG–RGD	MiR-200c	Liang et al., 2017 [74]
	Exosome	miR-21-3p	Zhang et al., 2021 [76]

## Data Availability

Not applicable.

## Abbreviations

NPs	Nanoparticles
MRI	Magnetic resonance imaging
PTT	Photothermal therapy
GnRH	Gonadotropin-releasing hormone
LH	Luteinizing hormone
FSH	Follicle-stimulating hormone
ROS	Reactive oxygen species
PPIX	Protoporphyrin IX
ALA	5-aminolevulinic acid
NIR	Near-infrared
FDA	Food and Drug Administration
ICG	Indocyanine green
SiNc-NP	Silicon naphthalocyanine-loaded polyethylene glycol-polycaprolactone NP
USPIO	Ultrasmall superparamagnetic iron oxide
HA	Hyaluronic acid
VEGFR-2	Vascular endothelial growth factor receptor-2
CA125	Cancer antigen 125
MWCNT	Multiwalled carbon nanotubes
CS	Chitosan
CA 19-9	Cancer antigen 19-9
AuNPs	Gold NPs
GO	Graphene oxide
GCE	Glassy carbon electrodes
HP	Haptoglobin
CNP	Cerium oxide NPs
OS	Oxidative stress
EGCG	Epigallocatechin gallate
DOX	Doxycycline
PLGA	Poly (lactic-co-glycolic acid)
MMP	Matrix metalloproteinase
CPO	Copaiba oleoresin
PCL	Poly ε-caprolactone
PEG	Polyethylene glycol
PDT	Photodynamic therapy
HAuNS	Hollow gold nanoshells
CuS	Copper sulfide
CTLA-4	Cytotoxic T lymphocyte antigen 4
PLGA	Poly (lactic-co-glycolic acid)
UNPs	Unmodified mesoporous silica NPs
AMNPs	Aminopropyl-modified silica NPs
GMDP	Glucosaminyl muramyl dipeptide
MMP-9	Matrix metalloproteinase 9
M1NVs	M1-like macrophages
EVs	Extracellular vesicles
AMF	Alternating magnetic field
MNs	Magnetic NPs
DNA	Deoxyribonucleic acid
CSO-SA/PEDF	Chitosan micelles/pigment epithelium-derived factor
PAMAM	Polyamidoamine
GFP	Green fluorescent protein
PEI-PEG-RGD	Polyethylenimine-polyethylene glycol-arginine-glycine-aspartic acid
miRNA@PEI-PEG-RGD	miR-200c mimic RNA Polyethylenimine -polyethylene glycol-arginine-glycine-aspartic acid
HESC’s	Human endometriotic stromal cells
ZEB1	Zinc finger E-box-binding homeobox 1
ZEB2	Zinc finger E-box-binding homeobox 2
qRT-PCR	Real-Time Quantitative Reverse Transcription PCR
MALAT1	Metastasis-associated lung adenocarcinoma transcript 1
